# Effects of Cyclosporine on Reperfusion Injury in Patients: A Meta-Analysis of Randomized Controlled Trials

**DOI:** 10.1155/2015/287058

**Published:** 2015-06-16

**Authors:** Kangxing Song, Shuxia Wang, Dake Qi

**Affiliations:** ^1^Department of Cardiology, The General Hospital of Chinese People's Liberation Army, No. 28, Fuxing Road, Beijing 100853, China; ^2^Department of Geriatric Cardiology, The General Hospital of Chinese People's Liberation Army, No. 28, Fuxing Road, Beijing 100853, China; ^3^Yale Cardiovascular Research Center, Department of Internal Medicine, Yale University School of Medicine, New Haven, CT 06511, USA

## Abstract

Mitochondrial permeability transition pore (mPTP) opening due to its role in regulating ROS generation contributes to cardiac reperfusion injury. In animals, cyclosporine (cyclosporine A, CsA), an inhibitor of mPTP, has been found to prevent reperfusion injury following acute myocardial infarction. However, the effects of CsA in reperfusion injury in clinical patients are not elucidated. We performed a meta-analysis using published clinical studies and electronic databases. Relevant data were extracted using standardized algorithms and additional data were obtained directly from investigators as indicated. Five randomized controlled blind trials were included in our meta-analysis. The clinical outcomes including infarct size (SMD: −0.41; 95% CI: −0.81, 0.01; *P* = 0.058), left ventricular ejection fraction (LVEF) (SMD: 0.20; 95% CI: −0.02, 0.42; *P* = 0.079), troponin I (TnI) (SMD: −0.21; 95% CI: −0.49, 0.07; *P* = 0.149), creatine kinase (CK) (SMD: −0.32; 95% CI: −0.98, 0.35; *P* = 0.352), and creatine kinase-MB isoenzyme (CK-MB) (SMD: −0.06; 95% CI: −0.35, 0.23; *P* = 0.689) suggested that there is no significant difference on cardiac function and injury with or without CsA treatment. Our results indicated that, unlike the positive effects of CsA in animal models, CsA administration may not protect heart from reperfusion injury in clinical patients with myocardial infarction.

## 1. Introduction

Ischemic heart disease is a leading cause of death and disability worldwide and it would continue to be the top reason of death till 2030 in both developing and developed countries [1]. Reperfusion therapy is the most important strategy to rescue ischemic myocardium and reduce infarction size [2]. However, it is often associated with a further cardiac injury functionally characterized by myocardial stunning, ventricular arrhythmias, and no-reflow [3, 4]. In histology, reperfusion injury leads to cell death in heart due to poor calcium handling in the sarcoplasmic reticulum-mitochondria system, calpain activation, oxidative stress, and mitochondrial damage [5, 6].

Mitochondrial dysfunction is a major factor leading to loss of cardiomyocyte function and viability [7]. The molecular mechanisms of mitochondrial dysfunction include a long-lasting opening of mitochondrial permeability transition pore (mPTPs) and oxidative stress resulting from reactive oxygen species (ROS) formation [6]. More recent studies indicated that mPTPs opening plays a crucial role in reperfusion injury [8]. The mPTPs opening during reperfusion could be regulated by several factors including high pH, Ca^2+^ overload, and burst of ROS at the onset of reperfusion [6, 9].

Cyclosporine, also known as cyclosporine A (CsA), is an inhibitor of mPTP opening, which has been proposed to prevent reperfusion injury following acute myocardial infarction [3, 10]. Some previous meta-analysis based on animal studies indicated that CsA might reduce myocardial infarct size [11]. However, the effects of CsA in clinical patients are largely unknown. A small pilot trial showed that administration of CsA at the time of percutaneous coronary intervention (PCI) limited infarct size during acute myocardial infarction, suggesting a positive effect of CsA in reperfusion injury [12]. However, another study with a similar number of patients reported that CsA treatment did not produce any beneficial effect on either infarct size or other clinical outcomes [13].

So far based on any individual human study, it is hard to draw a conclusion that CsA benefits reperfusion injury due to limited patient number and race. Therefore, our present analysis was to identify and combine all published clinical trials that investigated CsA treatment in human patients. We hope to see a clearer picture about the therapeutic effects of CsA on reperfusion injury.

## 2. Methods

### 2.1. Literature Screening

We systematically searched PubMed, Cochrane Library, Embase, reviews, and reference lists of relevant papers before September 2014 by using Medical Subject Heading (MeSH) terms “Cyclosporine,” “CSA” paired with the following terms: “reperfusion injury,” “reperfusion,” and “injury.”

### 2.2. Study Selection

Studies were selected based on the following criteria: (1) randomized controlled studies; (2) patients with myocardial infarction or with aortic valve surgery or with coronary artery bypass graft surgery (CABG); (3) CsA treatment.

### 2.3. Quality Assessment

All the recruited studies were assessed and scored by the following five indicators including the quality of randomization, blinding, reporting of withdrawals, generation of random numbers, and concealment of allocation. The possible score is from 0 to 5 and score 5 represents the highest level of quality [14].

### 2.4. Data Extraction

The data extracted from each study include the first-author's name, year of publication, country, subject characteristics (race, gender, age, and so on), sample size, and endpoints of patients. Cardiac injury following infarction was evaluated by magnetic resonance imaging (MRI), release of cardiac enzymes (troponin I, creatine kinase, and creatine kinase-MB isoenzyme), and left ventricular ejection fraction (LVEF) performed with two independent investigators.

### 2.5. Statistics and Analysis

All the data were presented as mean ± standard deviation (SD). The value of SD was calculated from published data in the original articles. Briefly, a random effect model was performed in the data with *P* value less than 0.10 by heterogeneity test (*χ* ^2^-based *Q*-test), while a fixed-effect model was used when *P* value is higher than 0.10 [15]. Heterogeneity was also assessed by *I* ^2^ test. The *I* ^2^ value was classified by the percentage of observed study variability based on heterogeneity rather than chance (*I* ^2^ = 0–25%, no heterogeneity; *I* ^2^ = 25–50%, moderate heterogeneity; *I* ^2^ = 50–75%, large heterogeneity; *I* ^2^ = 75–100%, extreme heterogeneity) [16]. Since CK values have significant heterogeneity, the random effect models were performed. In addition, funnel plots were used to assess publication bias. Statistical analysis was carried out with Stata software (version 12.0; Stata Corporation, College Station, TX) and REVMAN software (version 5.0; Cochrane Collaboration, Oxford, UK).

## 3. Results

### 3.1. The Data Extracting from Literatures

**Figure 1:**
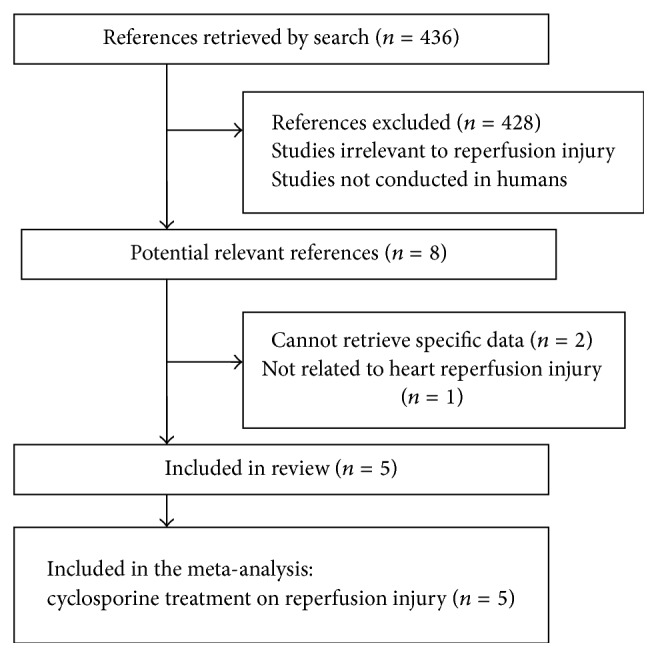
The flowchart outlining the process of search criteria and study selection.

A total of 436 articles were selected through the search and 428 of them were excluded due to the reasons like being irrelevant to reperfusion injury or nonhuman studies. A full text assessment in the eight potentially relevant articles led to a further exclusion of 3 studies (Figure 1). The exclusion was due to (1) failure to retrieve specific data for our meta-analysis [17, 18]; (2) being irrelevant to heart reperfusion injury [19]; or (3) lack of clinical data about left ventricular systolic dysfunction.

### 3.2. The Characteristics of Selected Studies

In the five recruited literatures [12, 13, 20–22], three studied patients with myocardial infarction [12, 13, 20], one studied patients following aortic valve surgery [21], and one studied patients following CABG surgery [22]. The CsA was given with an intravenous bolus dose (2.5 mg/kg) in all these studies. In addition, two studies were double-blinded [13, 22] and the other three were single-blinded [12, 20, 21]. The average age of patients varied from 58 to 67 years. The major characteristics of the selected studies have been listed in Table 1.

### 3.3. Data Quality

The quality scores of the trials varied from 3 to 5 (maximum score). All included trials were randomized, prospective, and placebo-controlled and blind design.

### 3.4. The Evaluation of CsA Effects on Clinical Outcomes

The clinical indicators including infarct size, left ventricular ejection fraction (LVEF), troponin I (TnI), creatine kinase (CK), and creatine kinase-MB isoenzyme (CK-MB) were analyzed in our data.

#### 3.4.1. Infarct Size

**Figure 2:**
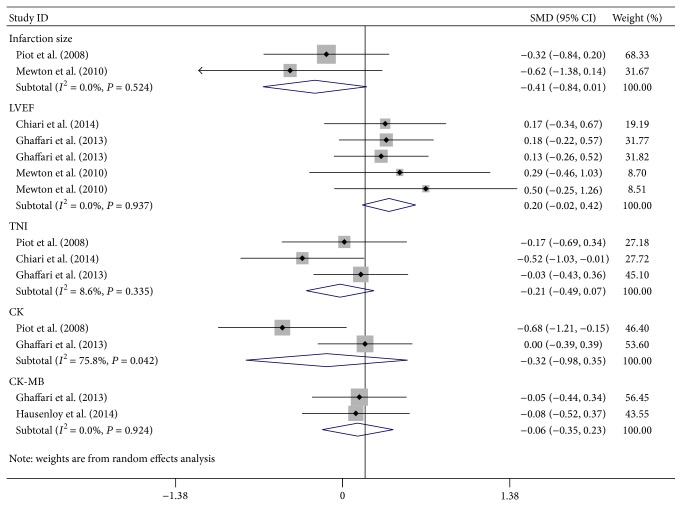
Random effect meta-analysis of standard mean differences (95% CI) on cardiac injury following cyclosporine treatment. The cardiac injury following reperfusion was quantified by infarct size, left ventricular ejection fraction (LVEF), creatine kinase (CK), and creatine kinase-MB (CK-MB) with and without cyclosporine treatment. The meta-analysis was performed on these data. Significance is *P* < 0.05.

Infarct size was measured by MRI in one study, which was evaluated twice at the fifth day [12] and 6 months [20], respectively, following myocardial infarction. Myocardial infarction was identified by delayed hyperenhancement within myocardium and quantified by intensity of myocardial postcontrast signal. The significance was defined by more than two SD above that in a reference region of remote, noninfarct myocardium within the same slice [12]. The CsA treatment group appears to have a smaller infarct size compared to control group, but the difference is not statistically significant (SMD: −0.41; 95% CI: −0.81, 0.01; *P* = 0.058) (Figure 2). In addition, no significant calculated heterogeneity (heterogeneity *χ* ^2^ = 0.41, *I* ^2^ = 0%, and *P*_heterogeneity_ = 0.52) was found (Figure 2).

#### 3.4.2. LVEF

LVEF was evaluated in three studies. One of them was measured at 5 days and 6 months following occurrence of myocardial infarction [20]. The second was assessed at the first day of admission and after hospital discharge [13]. The third was measured at hospital discharge [21]. There was no significant improvement on LVEF in CsA treatment group compared to control (SMD: 0.20; 95% CI: −0.02, 0.42; *P* = 0.079) (Figure 2). No significant heterogeneity in LVEF (heterogeneity *χ* ^2^ = 0.41, *I* ^2^ = 0%, and *P*_heterogeneity_ = 0.52) was found either (Figure 2).

#### 3.4.3. TnI

TnI was also quantified in three studies. The plasma level of TnI following CsA treatment (SMD: −0.21; 95% CI: −0.49, 0.07; *P* = 0.149) was comparable to control group (Figure 2) and the heterogeneity of TnI was also unchanged (heterogeneity *χ* ^2^ = 2.19, *I* ^2^ = 8.6%, and *P*_heterogeneity_ = 0.34) (Figure 2).

#### 3.4.4. CK and CK-MB

CK and CK-MB were evaluated in two studies, but none of them demonstrated changes following CsA treatment (CK: SMD: −0.32; 95% CI: −0.98, 0.35; *P* = 0.352) (CK-MB: SMD: −0.06; 95% CI: −0.35, 0.23; *P* = 0.689) (Figure 2). Interestingly, although a significant heterogeneity was observed in CK levels (heterogeneity *χ* ^2^ = 4.13, *I* ^2^ = 75.8%, and *P*_heterogeneity_ = 0.042), the heterogeneity of CK-MB was unchanged following CsA treatment (heterogeneity *χ* ^2^ = 0.01, *I* ^2^ = 0%, and *P*_heterogeneity_ = 0.924). The random effect models were subsequently performed to attenuate the effects of CK heterogeneity between the two studies.

### 3.5. Publication Bias

**Figure 3:**
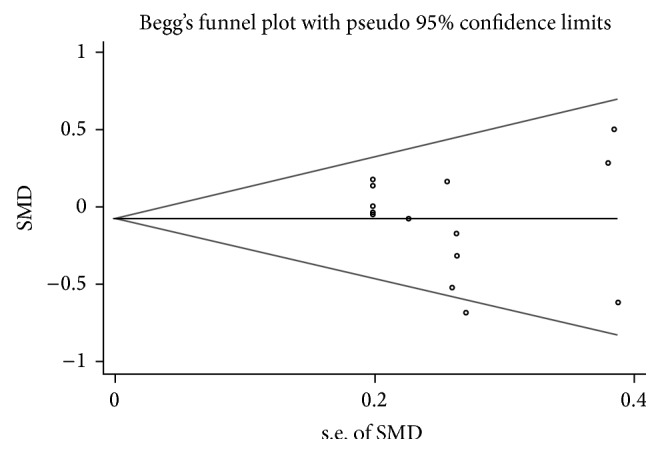
Begg's funnel plot (with pseudo 95% CIs) of all studies included in the meta-analysis.

Funnel plots of the study are symmetric through visual examination and a statistical analysis of funnel plots also suggested there is no publication bias (Egger test, *P* = 0.44) (Figure 3).

## 4. Discussion

Reperfusion injury is associated with numerous cellular mechanisms including inflammatory responses, vascular leakage, free radical generation, and proapoptotic protein release [8]. Mitochondrial permeability transition pore (mPTP) plays a key role in regulating cell death and oxidative stress [8]. The mPTP opening allows small molecular solutes to freely move into the mitochondrial matrix leading to resultant mitochondrial swelling. This swelling then causes unfolding of cristae and release of intermembrane proteins that eventually trigger cell apoptosis [23, 24]. Therefore, the mPTP opening may significantly contribute to cardiac reperfusion injury. Indeed, some previous studies have indicated that mPTP inhibitor, CsA, had a positive effect on reperfusion injury in animal models [25, 26]. The treatment of CsA in old rats is associated with decreased oxidative stress and improved mitochondrial function [27]. However, whether the inhibition of mPTP opening is also beneficial to ischemia-reperfusion injury in human being is still controversial.

In our meta-analysis, the five recruited studies have a randomized, controlled, and blind design. All the patients were intravenously injected with a bolus dose of CsA at 2.5 mg/kg before PCI, cardiac surgery, or thrombolytic treatment. Our aim was to evaluate the effects of CsA on reperfusion injury in human patients. Infarct size is a key indicator for postischemic injury in the heart and it is also a marker for cell death [28]. Cell death during reperfusion is associated with preapoptotic pathway c-Jun N terminal kinase (JNK) activation [29]. The mPTP opening facilitates ROS generation, which is a trigger of JNK upstream kinases [30]. Simultaneously, the increased JNK activation further upregulates ROS production [31]. Therefore, CsA administration may inhibit reperfusion injury through downregulating ROS production and JNK pathway activation. In our current analysis, two selected studies measured infarct size by MRI at early (the fifth day after acute myocardial infarction) [12] and late reperfusion (6 months) [20]. The group with CsA treatment demonstrated a similar infarct size as control at both time points, suggesting that CsA regulated mitochondrial function is not a mechanism to stop the development of cell necrosis during reperfusion in the heart.

Cell necrosis leads to intracellular content release. Therefore, some cardiac specific enzymes including TnI, CK, and CK-MB have been widely used in clinical practice to identify cardiac injury. Our results indicated that CsA treatment did not alter TnI in clinical patients receiving PCI, CABG, or aortic valve surgery. However, plasma CK has a significant heterogeneity. We usually use the subgroup and metaregression analysis to explore heterogeneity in effects and influences of study characteristics or perform a random effect model to attenuate the effects of heterogeneity. In our current study, the CK analysis due to its small sample size is hard to run either subgroup or metaregression analysis. Random effect models assume that the treatment effects observed in the trials are random samples from a distribution of treatment effects with heterogeneity which typically produce more conservative estimates of the significance of the treatment effect than fixed-effect models [32]. Therefore, the random effect models were performed to reduce the effects of heterogeneity in our small sample studies. Eventually, our results indicated that plasma CK is not changed following CsA treatment, suggesting that CsA treatment did not affect reperfusion injury.

Overall, our current meta-analysis indicated that CsA may not protect heart from reperfusion injury in clinical patients. This conclusion is based on the tests from a small group of clinical patients. The restricted data leads to a result that no further subanalysis could be performed in patients with acute myocardial infarction or aortic valve surgery. In addition, a major confounding factor is the heterogeneity among cases and controls. Therefore, more careful selections of cases and controls in larger studies will be required to firmly establish the role of CsA in regulating reperfusion injury.

## Figures and Tables

**Table 1:** Characteristics of the study population.

Reference	Year	Cyclosporine method	Cyclosporine dosage	Participants	Number of subjects	Age	Study design
Chiari et al. [21]	2014	Intravenous bolus	2.5 mg/kg	Patients accepting elective aortic valve surgery	30	67 ± 11	Prospective, monocentric, randomized, controlled, single-blind
Ghaffari et al. [13]	2013	Intravenous bolus	2.5 mg/kg	Patients with acute anterior STEMI receiving TLT	50	64.0 ± 11.2	Randomized, placebo-controlled, double-blinded
Hausenloy et al. [22]	2014	Intravenous bolus	2.5 mg/kg	Patients undergoing elective CABG surgery [1]	40	65.8 ± 10.7	Randomized, placebo-controlled, double-blinded
Mewton et al. [20]	2010	Intravenous bolus	2.5 mg/kg	Patients with AMI accepting PCI	15	60 ± 10	Prospective, multicenter, randomized, controlled, single-blind
Piot et al. [12]	2008	Intravenous bolus	2.5 mg/kg	Patients with [2] AMI accepting PCI	30	58 ± 2	Prospective, multicenter, randomized, controlled, single-blind

STEMI: ST-elevation myocardial infarction; CABG: coronary artery bypass graft; AMI: acute myocardial infarction; PCI: percutaneous coronary intervention; TLT: thrombolytic treatment.
